# iCap: Instrumented assessment of physical capability

**DOI:** 10.1016/j.maturitas.2015.04.003

**Published:** 2015-09

**Authors:** A. Godfrey, J. Lara, S. Del Din, A. Hickey, C.A. Munro, C. Wiuff, S.A. Chowdhury, J.C. Mathers, L. Rochester

**Affiliations:** a Institute of Neuroscience, Newcastle University, Campus for Ageing & Vitality, Newcastle upon Tyne, UK; b Clinical Ageing Research Unit, Newcastle University, Campus for Ageing & Vitality, Newcastle upon Tyne, UK; c Institute of Cellular Medicine, Newcastle University, Campus for Ageing & Vitality, Newcastle upon Tyne, UK; d Human Nutrition Research Centre, Newcastle University, Campus for Ageing & Vitality, Newcastle upon Tyne, UK

**Keywords:** Accelerometer, Gait speed, Endurance, TUG, Standing balance, Lower limb strength

## Abstract

**Objectives:**

The aims of this study were to (i) investigate instrumented physical capability (iCap) as a valid method during a large study and (ii) determine whether iCap can provide important additional features of postural control and gait to categorise cohorts not previously possible with manual recordings.

**Study design:**

Cross-sectional analysis involving instrumented testing on 74 adults who were recruited as part of a pilot intervention study; LiveWell. Participants wore a single accelerometer-based monitor (lower back) during standardised physical capability tests so that outcomes could be compared directly with manual recordings (stopwatch and measurement tape) made concurrently.

**Main outcome measures:**

Time, distance, postural control and gait characteristics.

**Results:**

Agreement between manual and iCap ranged from moderate to excellent (0.649–0.983) with mean differences between methods low and deemed acceptable. Additionally, iCap successfully quantified (i) postural control characteristics which showed sensitivity to distinguish between 5 variations of the standing balance test and (ii) 14 gait characteristics known to be sensitive to age/pathology.

**Conclusions:**

Our findings show that iCap can provide robust quantitative data about physical capability during standardised tests while also providing sensitive (age/pathology) postural control and gait characteristics not previously quantifiable with manual recordings. The methodology which we propose may have practical utility in a wide range of clinical and public health surveys and studies, including intervention studies, where assessment could be undertaken within diverse settings. This will need to be tested in further validation studies in a wider range of settings.

## Highlights

• Instrumented testing of five physical capability tasks with a single accelerometer.
• Evaluated on a large cohort of older adults.
• iCap provides robust quantitative data about physical capability.
• iCap captures gait and postural control data known as sensitive to ageing/pathology.
• Methodology may have practical utility in a wide range of surveys and studies.

## 1 Introduction

Maintenance of good health is an important foundation for ageing well [1] because poor health disrupts daily life and reduces the ability to manage the activities of daily living [2]. Physical capability (defined as the physical/functional capacity of an individual to carry out successfully the activities of everyday life) is an important objective measure of health [3,4]. Moreover, there is a growing interest in epidemiological and intervention studies focusing on age-related change in physical capability which aim to characterise ageing using a battery of surrogate markers of the ageing process [5,6]. Capturing physical capability quantitatively is therefore central to operationalization of the ageing process and could also prove useful as an outcome measure in future studies [5].

Early attempts to quantify physical capability used questionnaire based assessments [7,8]. More recently, a battery of measures has been proposed to capture physical capability and has been shown to predict health in later life. These include: postural control, gait (speed and endurance), lower limb strength and locomotion (timed-up-and-go, TUG) [3–5]. These measures are proposed because they are simple and convenient for implementation in any environment and can be measured in a standardised manner. However, variations in testing protocols and rater reliability have been raised as issues which may limit the ability to pool data across multicentre studies [3,4]. For example, some physical capability outcome measures are quantified using a stop-watch. Potential limitations of these manual methods include accurate identification of the beginning and end of a test (such as moving from sitting to standing) which can lead to heterogeneity of reported outcomes [9]. Capturing millisecond changes in postural control or characteristics of gait (which have been shown to be sensitive to ageing/pathology) is also impossible with a stop-watch [10–12].

Inconsistent application and reporting have therefore led to efforts to harmonise protocols and measures to facilitate data capture, reliability, and data pooling across trials [5]. One potential solution to overcome some of these limitations is to instrument tests using accelerometer-based body worn monitors (BWM) and to adopt standardised protocols [5,6,13] as recommended by the NIH Toolbox [14,15]. To date, simultaneous instrumentation of testing protocols has not been adopted. However, a recent study has described and established that it is feasible to fuse a number of tailored algorithms for use in a single BWM to quantify tasks relating to physical capability [16]. Here we applied this novel approach to simultaneously instrument a battery of recommended and validated physical capability (iCap) tests [3,4,14] in a large sample of adults.

Therefore, the first aim of this study was to compare measures derived from iCap with those from a stopwatch to establish agreement between the approaches. Secondly, we report additional outcomes possible only with a BWM (iCap plus) to explore advantages of an instrumented approach. In this study we adopted standardised and validated protocols/test [5] to evaluate postural control and report postural control characteristics which have been identified as sensitive to ageing/pathology [10,17] and compared sensitivity of accelerometer-derived measures with respect to task difficulty. Finally we determined a battery of validated gait characteristics [12] collected during the endurance task also described as sensitive to ageing/pathology [18,19]. The proposed methodology (adoption of standardised tests and iCap) may have practical utility in a wide range of clinical and public health surveys/studies (including interventions) where assessment/data could be conducted/collected and compared across many settings.

## 2 Methodology

### 2.1 Participant recruitment and measurement

Participants were recruited in the North East of England as part of a pilot study^2^ within the LiveWell programme. Inclusion criteria consisted of: aged 50–70 years, community dwelling, male or female, physically capable (i.e. no neurological conditions that might affect their gait or balance), regular internet users, English language speakers and in the retirement transition (approximately 2 years before/after retirement). Ethical consent for the project was granted by the Newcastle University Faculty of Medical Sciences ethics committee (00745/2014) and all participants gave informed written consent. Participant recruitment was arranged through large employers on Teesside and on Tyneside.

Standardised anthropometric measurements were taken in private in the leisure centre facilities of each community. Body weight, height, and waist circumference, were measured using standard methods [20]. Briefly, body weight was recorded to the nearest 100 g, in all subjects without shoes and wearing light clothing using a scale (Tanita 300). Height was measured in metres with subjects wearing light clothing and without shoes, using a portable Leicester height measure device. BMI was calculated as weight (kg)/(height (m))^2^.

### 2.2 Equipment

**Fig. 1:**
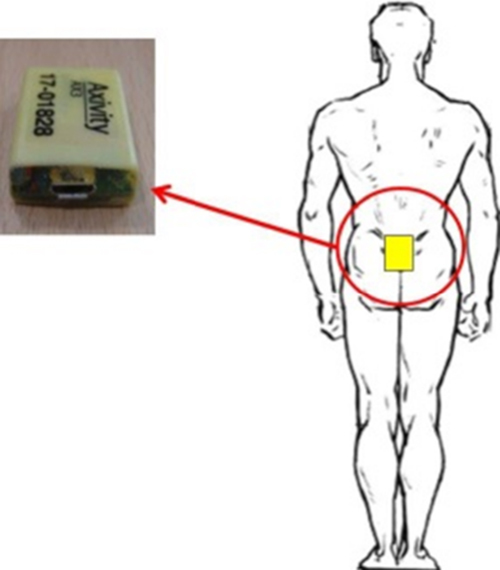
Attachment of the BWM to the lower back (L5).

Each participant wore a low cost tri-axial accelerometer-based BWM (Axivity AX3, York, UK, dimensions: 2.3 cm × 3.3 cm × 0.8 cm, weight 9 g: sampling frequency 100-Hz, resolution: 16-bit, range: ±8 g) on the fifth lumbar vertebrae (L5), Fig. 1. This location was chosen to minimise problems with device attachment during instrumented testing while also optimising algorithm usage, i.e. numerous algorithms developed for use on L5. The BWM was held in place by double sided tape and Hypafix (BSN Medical Limited, Hull, UK). A trained researcher used a stop-watch and measurement tape (as appropriate) to record outcomes for each standardised physical capability task.

### 2.3 Experimental protocol (iCap)

A battery of validated physical capability tests [5] was conducted and data were collected simultaneously using a BWM and manual recordings (where appropriate). In Tyneside, testing took place at Newcastle University facilities, while in Teesside testing was carried out at community leisure centres. The assessment comprised the following tests which were applied in a non-randomised order:

(i) Locomotion – 4-m walk gait speed (×2): after a practice walk, participants walked at their usual speed between 2 markers. Manual and iCap timing began on the first footfall, i.e. participant's first step over the starting point. Recording ended after the participant completed the walk (manual) or last ‘purposeful’ footfall as determined by iCap [16,21]. Time to complete the 4 m walk was converted into a metres-per-second metric and averaged between trials:
  $$\text{Speed}(\text{m}/\text{s})=\frac{\text{Distance}(4\text{m})}{\text{Time}(\text{time}\;\text{to}\;\text{walk}\;4\text{m})}$$
(ii) Lower limb strength – repeated sit-to-stand-to-sit (×2): after a practice, participants performed 5 sit-to-stand-to-sit posture transitions (PT), with arms folded across their chest, as quickly as possible. Participants were instructed to stand fully and not to touch the back of the chair during each repetition. Average time to complete both trials is presented.
(iii) Lower limb strength with locomotion – TUG (×3): after a practice, participants stood up from a chair (height: 40–50 cm), walked 2 m at a normal pace, around a cone, back to the chair, turned and sat down. The TUG time was recorded manually as the time from initiation of chair rise to the time when the participant's back touched the backrest of the chair at the end of the manoeuvre. The average time across the three trials is presented.
(iv) Postural control – standing balance: 5 tests were performed each lasting 50 s without shoes, arms folded across participant's chest, focusing on a wall-mounted fixed point (target) at a horizontal distance of 1 m. Variations included: (i) flat surface, feet together, eyes open (FLFTEO), (ii) flat surface, feet together, eyes closed (FLFTEC), (iii) foam surface^3^ (50.0 cm × 41.0 cm × 6.0 cm), feet together, eyes open (FOFTEO), (iv) foam surface, feet together, eyes closed (FOFTEC) and (v) flat surface, tandem stance, eyes open (FLTMEO). BWM-based characteristics such as magnitude and frequencies were quantified for each test, Section 2.4.
(v) Endurance – 2-min walk: participants walked continuously and as fast as they could without running. The route consisted of walking back and forth around cones placed 25 ft (7.62 m) apart. Once completed, the total distance walked was calculated manually. In addition, 14 gait characteristics sensitive to age/pathology were quantified by the BWM [18,19] during the duration of this test.

### 2.4 BWM algorithms

The algorithms for iCap have been described previously [16]. In brief:

(i) Algorithm #1 (locomotion/endurance): a continuous wavelet transform estimated the initial (IC) and final contact (FC) gait events [21]. Subsequently, the IC/FC times were used to record total time to complete the 4 m test as well as step, stride, stance and swing times.
(ii) Algorithm #2 (lower extremity strength, TUG): PT and TUG were estimated from a refined version [16] of a discrete wavelet transform based on the combination of tri-axial accelerometer data and peak/trough recognition [22].
(iii) Algorithm #3 (postural control): Jerk (rate of change of acceleration), root mean square (RMS, magnitude) and frequency components (95% percentile (F95%), ellipsis) were evaluated [10,17]. Due to its sensitivity, we present data within the mediolateral (ML) direction only [17]. (However, this methodology can also be applied to the AP and combined directions [10,17].)
(iv) Algorithm #4 (endurance): complementary to the IC/FC algorithm, we applied the inverted pendulum model [23] to estimate step length and hence total distance walked during the endurance test.

Algorithm #1 + #4 (endurance): the estimates of step time and length were combined to generate values for step velocity [16].

### 2.5 Statistical analysis

Normality of data distributions were tested using a Shapiro–Wilk test with descriptives presented as mean (±standard deviations, SD) or median (range) values. Levels of agreement (LoA) between the manual reference methods and iCap were expressed as interclass correlation coefficients (ICCs) of type (2, *k*) and as mean differences ($\bar{x}$) ± 2 SD (95% LoA) [24]. A Friedman test with Bonferroni correction for pairwise (post hoc) comparisons was used to examine differences in postural control with respect to task difficulty. Statistical significance was set at *p* < 0.05 (unless stated otherwise) with acceptance ratings for ICCs set at excellent (>0.900), good (0.750–0.899), moderate (0.500–0.749) and poor (<0.500) [25,26]. Analysis was performed using SPSS^4^ v21.

## 3 Results

Seventy-five participants were recruited and their demographics are presented in Table 1. More women were recruited (ratio 3:1) which is common in lifestyle interventions [27] with an average age for all participants of 61 years. BMI was similar to national values [28] and normal for 38% of the participants (*n* = 28) and >60% were overweight (*n* = 31) or obese (*n* = 15), Table 1.

### 3.1 iCap and manual recording agreement

**Fig. 2:**
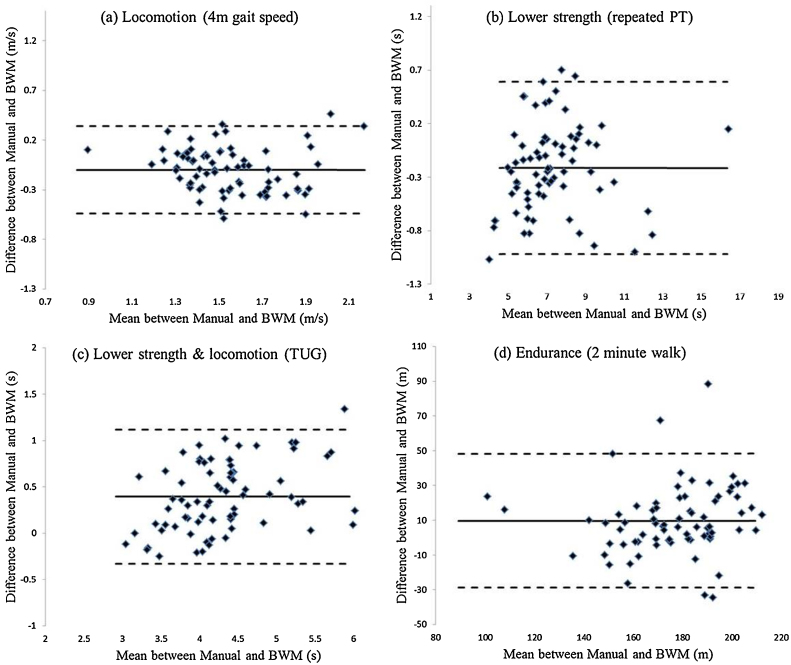
Bland–Altman plots of physical capability tasks between manual and BWM methods. Solid line systematic bias; dashed lines represent 95% LoA (±SD × 1.96).

Table 2 and Fig. 2(a–c) show good/excellent agreement between manual and iCap estimates of the 4 m gait speed (locomotion, ICC = 0.759), repeated sit-to-stand-to-sit PT (lower limb strength, ICC = 0.983) and TUG (lower limb strength with locomotion, ICC = 0.926). Mean differences were low with iCap recording slightly lower (faster) values for TUG (<0.4 s) and greater (slower) values for gait speed (0.1 m/s) and repeated PT (approx. 0.2 s) than manual estimates. Agreement for total distance measured during 2 min walk (endurance) was moderate (ICC = 0.649) with iCap recording greater (longer) distances by approximately 9.5 m, Table 2 and Fig. 2(d).

### 3.2 iCap plus: postural control characteristics (standing balance)

The standing balance test was used to extract information on characteristics of postural control. Jerk_ML_ (main effect, *χ*^2^(4) = 189.914), RMS_ML_ (main effect, *χ*^2^(4) = 178.627), ellipsis (main effect, *χ*^2^(4) = 172.173) and F95%_ML_ (main effect, *χ*^2^(4) = 47.889) were significantly different between all conditions (*p* < 0.0005), Table 3. Increasing complexity of standing balance task (flat surface to foam or eyes open to closed) resulted in increasing postural control values for Jerk_ML_, RMS_ML_ and ellipsis but the opposite was observed for F95%_ML_, Table 3.

### 3.3 iCap plus: gait characteristics (endurance)

In addition to total distance walked in 2 min, we quantified 14 previously validated [12] gait characteristics (*n* = 66) relating to spatio-temporal performance, variability and asymmetry known to be sensitive to ageing/pathology [18,19], Table 4. They generally show high level of performance in this group [29,30].

Data for 8 participants with extreme outliers (values >3 box lengths from edge of boxplot, SPSS) were removed from this analysis due to very unusual values encountered for all characteristics. Examination of these outliers revealed no bias for age (range: 57–70 years), BMI (range: 20.50–42.11 kg/m^2^) or gender (2M/6F) given the ratio of men to women recruited, suggesting algorithm limitations rather than participant characteristics with abnormal values.

## 4 Discussion

This study tested the use of an instrumented physical capability (iCap) assessment in a large cohort of adults. In addition to estimating objective physical capability outcomes, iCap provided gait and postural control characteristics not previously quantifiable during traditional physical capability assessments. Our findings suggest that this methodology may have practical utility in a wide range of clinical and public health surveys and studies, including intervention studies, where it may facilitate physical capability assessment within many settings. With a growing interest in the identification and development of objective (bio) markers of ageing capable of predicting ageing-related phenotypes (e.g. morbidity, mortality, quality of life or health span), and amenable to modification by lifestyle interventions, the usefulness of more detailed characterisation of gait and of postural control as potential objective markers of ageing should be evaluated in longitudinal studies of ageing.

### 4.1 Validation of iCap

The monitor adopted in this study is a generic device which allows access to the raw acceleration data which registers movement and subsequent implementation of appropriate algorithms [16]. iCap robustly quantified gait speed (ICC = 0.759) with little difference (0.1 m/s) compared with manual observations (Table 2). Gait speed (locomotion) is a strong predictor of longevity [9] and iCap facilitates its objective evaluation [16] with the LoA small enough for us to be confident that the method is reliable, Fig. 2(a). In addition, values are similar to those reported in adults [9]. Stringent application of standardised protocols will ensure accurate distance (4 m) and step count to further minimise any under/overestimation of gait speed due to observer or algorithm when instrumenting gait [12].

Repeated sit-to-stand-to-sit PT (lower extremity strength) resulted in excellent agreement (ICCs = 0.983) with manually recorded times. The enhanced accuracy for repeated PT was achieved through the adoption of a more suitable correction factor to account for the composite nature of the task [16]. iCap adopted the same algorithm from repeated PT within the TUG test (lower limb strength with locomotion) and we found excellent reliability (ICC = 0.926) and LoA without the need for any correction factors, Fig. 2(c). TUG times were lower (quicker) when estimated by iCap due to the definition of the TUG and algorithm functionality [16,31] but the differences in values between methods were within acceptable ranges (<0.4 s). Moreover, we can be confident of our instrumented TUG times based on a relative comparison to another instrumented study [32] that used a 7 m walk.

Agreement for total distance walked during the endurance task (2 min walk) was moderate and can be attributed to the nature of the walking protocol (walking back and forth incorporating abrupt directional changes). The algorithm which we used to derive distance walked is better suited to consistent straight line walking [23]. Moreover, it was observed that the scatter of the differences increases with increasing distance, Fig. 2(d). This implies that the LoA would be large for small distance but small for large distances. Given these findings it could be used as a suitable proxy for total distance during prolonged walks (>2 min).

Our results show that iCap may be a useful tool to measure physical capability as we found moderate to excellent agreement compared with manual recording by a trained researcher. Therefore, this method has potential as a low cost approach that could be adopted for widespread implementation in multi-centre studies to provide objective assessment and facilitate data pooling, a key recommendation for modern protocols [5]. However, the algorithms need to be evaluated longitudinally to examine their robustness in assessing the effects of ageing/pathology. Moreover the implementation of iCap requires data handling and processing expertise that goes beyond many clinical/epidemiological studies and therefore needs to be implemented within a user friendly software package.

### 4.2 iCap plus

iCap successfully quantified accelerometer-based postural control outcomes that have been shown to be better or consistent with centre of pressure outcomes quantified using traditional methods (i.e. force plates) [11]. These will be useful during longitudinal studies, examining effect of intervention [5] or disease progression in a pathological cohort [10]. We observed that all characteristics were sensitive to task difficulty, i.e. variation of standing Table 3. This is due to increased body sway and hence more movement detected by the BWM. A previous study detailed use of the iSway [10] to instrument postural control in a small sample of patients with Parkinson's disease and healthy controls and while it is difficult to compare our postural control results directly with iSway due to methodological differences (30 s test) we do observe similarities with their healthy cohort for estimates of Jerk, RMS and frequencies. Some studies show that RMS is sensitive to test conditions, ageing, and history of falls, while Jerk has been reported as the most discriminative measure to differentiate sway in patients with untreated Parkinson's disease [10]. However the applicability of these parameters to establish functional decline with ageing among healthy people needs further testing.

In addition iCap successfully quantified 14 gait characteristics known to be sensitive to age/pathology [18,19] during the 2 min walk, Table 4. We chose to quantify the gait characteristics during this prolonged gait activity (≥30 steps) to better assess variability/asymmetry [33] and found similar values to another study [12]. However, we observed eight extreme outliers in our data which can be attributed primarily to the protocol, i.e. walking back and forth around cones resulting in abrupt and extreme directional changes and consequently wide variation in gait characteristics, hence the reporting of their median/range values. However, when quantified during the shorter walks (4 m/TUG) and in comparison with a study of similar distance (3 m), and (healthy) cohort [34] we observed comparable values, lending confidence in the use of iCap to accurately quantify gait.

### 4.3 Use of existing technology and possible developments

We have shown that iCap is a robust methodology with potential for use in clinic and community environments, multicentre studies to improve consistency by reducing error from less experienced testers and offers the possibility for in home testing. We used a generic movement monitor but in the future this may be feasible with any modern media/communication device as they routinely integrate the appropriate sensors (accelerometers, gyroscopes).

## 5 Conclusion

Instrumented physical capability can be achieved robustly with a single tri-axial accelerometer-based BWM and appropriate algorithms. This approach also provides useful postural control and gait characteristics. Current algorithms require fixed BWM location but future developments could integrate the methodology within current technology (e.g. mobile phone) to ease user burden. The methodology which we propose may have practical utility in a wide range of clinical and public health surveys and studies, including intervention studies, where assessment/data could be conducted/collected and compared across many settings.

## Contributors

AG and LR conceived the methodology and drafted the paper with help from JL, JCM. JL, JCM, LR and AG developed the protocol. JL, CAM, CW and SAC conducted the assessments and gathered all the accelerometer and manual data. AG, SDD and AH scripted all the Matlab^®^ algorithms. All authors contributed to the critical revisions of the manuscript including analysis and interpretation of data.

## Competing interest

There is no conflict of interest.

## Funding

All authors acknowledge support from the LiveWell program a research project funded through a collaborative grant from the Lifelong Health and Wellbeing (LLHW) initiative, managed by the Medical Research Council (MRC) on behalf of the funders: Biotechnology and Biological Sciences Research Council, Engineering and Physical Sciences Research Council, Economic and Social Research Council, Medical Research Council, Chief Scientist Office of the Scottish Government Health Directorates, National Institute for Health Research (NIHR)/The Department of Health, The Health and Social Care Research & Development of the Public Health Agency (Northern Ireland), and Wales Office of Research and Development for Health and Social Care and the Welsh Assembly Government (grant number: G0900686). LR and AG are supported by the National Institute for Health Research (NIHR) Newcastle Biomedical Research Centre (BRC) and Unit (BRU) based at Newcastle upon Tyne Hospitals NHS Foundation Trust and Newcastle University. The research was also supported by NIHR Newcastle CRF Infrastructure Funding. The views expressed are those of the authors and not necessarily those of the NHS, NIHR or the Department of Health. SDD is supported by the V-Time project, which is a European Union 7th Framework Programme (FP7) under the Health theme (FP7: 278169).

## Ethical approval

Ethical consent for the project was granted by the Newcastle University Faculty of Medical Sciences ethics committee (00745/2014) and all participants gave informed written consent prior to data collection.

## Figures and Tables

**Table 1:** Demographic characteristics of the participants.

Characteristic	Mean ± SD
Gender (M/F)	16/58
Age (years)	61.30 ± 3.45
Height (m)	1.66 ± 0.09
Weight (kg)	73.53 ± 15.46
BMI (kg/m^2^)	26.79 ± 4.97
<25.0 (*n*)	28
25–29.9 (*n*)	31
30–34.9 (*n*)	10
≥35 (*n*)	5

**Table 2:** Mean values ±SD of the manual recorded values for locomotion (4 m gait speed), lower limb strength (sit-to-stand), lower limb strength with locomotion (TUG) and endurance (2 min walk) tasks. Also shown are the mean differences, 95% LoA and ICC values.

Task (*n* = 74)	Manual	BWM	Manual − BWM	
	Mean ± SD	Mean ± SD	$\bar{x}\pm 95\%$	ICC_(2,1)_
Locomotion (m/s) 4 m *gait speed*	1.50 ± 0.24	1.60 ± 0.26	−0.10 ± 0.45	0.759*
Lower limb strength (s) *Repeated sit-to-stand-to sit*	7.06 ± 1.78	7.40 ± 2.04	−0.21 ± 0.82	0.983*
TUG (s) *Lower limb strength & locomotion*	4.50 ± 0.77	4.11 ± 0.64	0.39 ± 0.74	0.926*
Endurance (m) 2 *min walk*	171.41 ± 22.19	181.08 ± 24.70	9.67 ± 39.33	0.649*

$\bar{x}$: mean differences.

* *p* < 0.001.

**Table 3:** Parameter estimates from postural control data obtained from the standing balance test.

Trial (*n* = 74)	Postural control characteristics – median (range)			
	Jerk_ML_(m^2^/s^5^)	RMS_ML_ (mm/s^2^)	Ellipsis (mm^2^)	F95%_ML_ (Hz)
(1) FLFTEO	0.017 (0.742)	0.008 (0.048)	0.073 (1.821)	2.030 (2.980)
(2) FLFTEC	0.028 (0.428)	0.009 (0.029)	0.096 (1.118)	1.900 (2.460)
(3) FOFTEO	0.041 (4.974)	0.010 (0.063)	0.128 (7.054)	1.810 (3.340)
(4) FOFTEC	0.227 (6.963)	0.019 (0.163)	0.671 (10.282)	1.660 (3.120)
(5) FLTMEO	0.054 (8.032)	0.011 (0.109)	0.130 (13.481)	2.260 (2.740)

FLFTEO: flat surface, feet together, eyes open; FLFTEC: flat surface, feet together, eyes closed; FOFTEO: foam surface, feet together, eyes open; FOFTEC: foam surface, feet together, eyes closed; FLTMEO: flat surface, tandem stance, eyes open.

**Table 4:** Estimates of spatio-temporal gait characteristics obtained from the 2 min walking test (8 extreme outliers removed from entire cohort of 74).

Task (*n* = 66)	Gait characteristic	Mean ± SD
Endurance	Step velocity (m/s)	1.539 ± 0.196
*2* *min walk*	Step length (m)	0.697 ± 0.081
	Step time (s)	0.459 ± 0.034
	Stance time (s)	0.589 ± 0.043
	Step length variability (m)	0.101 ± 0.022

Task (*n* = 66)	Gait characteristic	Median (Range)
Endurance	Swing time variability (s)	0.061 (0.129)
*2* *min walk*	Swing time (s)	0.330 (0.137)
	Step time variability (s)	0.062 (0.129)
	Step velocity variability (m/s)	0.222 (0.262)
	Stance time variability (s)	0.062 (0.128)
	Swing time asymmetry (s)	0.007 (0.033)
	Step time asymmetry (s)	0.007 (0.041)
	Stance time asymmetry (s)	0.006 (0.033)
	Step length asymmetry (m)	0.009 (0.060)

## Footnotes

2 Protocol registered at ClinicalTrials.gov (NCT02136381).

3 Balance-pad Elite, AIREX, Switzerland.

4 IBM SPSS Statistics, IBM Corporation, USA.
